# Genome Sequence of a Ranavirus Isolated from a Red-Eared Slider (Trachemys scripta elegans) in Poland

**DOI:** 10.1128/MRA.00781-20

**Published:** 2020-11-19

**Authors:** Ewa Borzym, Magdalena Stachnik, Michał Reichert, Artur Rzeżutka, Agnieszka Jasik, Thomas B. Waltzek, Kuttichantran Subramaniam

**Affiliations:** a Department of Fish Diseases, National Veterinary Research Institute, Pulawy, Poland; b Department of Food and Environmental Virology, National Veterinary Research Institute, Pulawy, Poland; c Department of Pathology, National Veterinary Research Institute, Pulawy, Poland; d Department of Infectious Diseases and Immunology, College of Veterinary Medicine, University of Florida, Gainesville, Florida, USA; e Emerging Pathogens Institute, University of Florida, Gainesville, Florida, USA; Portland State University

## Abstract

The red-eared slider (RES) ranavirus (RESRV) was isolated from a free-ranging RES turtle that died with evidence of respiratory disease. The RESRV genome sequence (106,878 bp) was determined, and phylogenetic analysis revealed that it is a common midwife toad virus (CMTV) strain. This study is the first report of CMTV in RES.

## ANNOUNCEMENT

Red-eared sliders (RES; Trachemys scripta elegans) are semiaquatic turtles with a native range extending from the southeastern United States to northern Mexico. They are listed as an invasive alien species (IAS) by the European Union legislation (1). A RES was captured from a shallow retention reservoir in Majdan Zahorodynski in eastern Poland (51°13′44″N, 23°08′26″E) and soon after died with signs of respiratory disease. A necropsy revealed congestion and edema with petechial hemorrhages in the tracheal mucosa and liver. Splenomegaly as well as hepatic and tracheal necrosis strongly suggested a viral infection. Homogenates prepared from the animal’s pooled tissues (e.g., liver, kidney, spleen) were used for virus isolation attempts in Terrapene carolina heart (TH-1) cells (CCLV-RIE 1131), as previously described (2). Cytopathic effects, including cell rounding, detachment, and lysis, were observed in the first cell passage. DNA was extracted from the cell culture supernatant using a Qiagen DNeasy blood and tissue kit, and the sample tested positive for ranavirus by quantitative PCR (qPCR) (3).

Viral DNA served as the template for constructing a DNA library using a TruSeq Dual Index high-throughput (HT) DNA PCR-free library preparation kit (Illumina), followed by sequencing on an Illumina MiSeq sequencer using a v3 chemistry 600-cycle kit. *De novo* assembly of the paired-end reads was performed in SPAdes v3.13.0 with default parameters (4). BLASTN analysis of the resulting three contigs was performed against the National Center for Biotechnology Information (NCBI) nonredundant nucleotide database. The largest contig was 106,878 bp, with a G+C content of 56% and an average coverage of 11,055 reads/nucleotide, and showed the highest nucleotide identity (98.85%) to a common midwife toad virus (CMTV) strain (Pelophylax esculentus virus [PEV]; GenBank accession no. MF538627).

**FIG 1:**
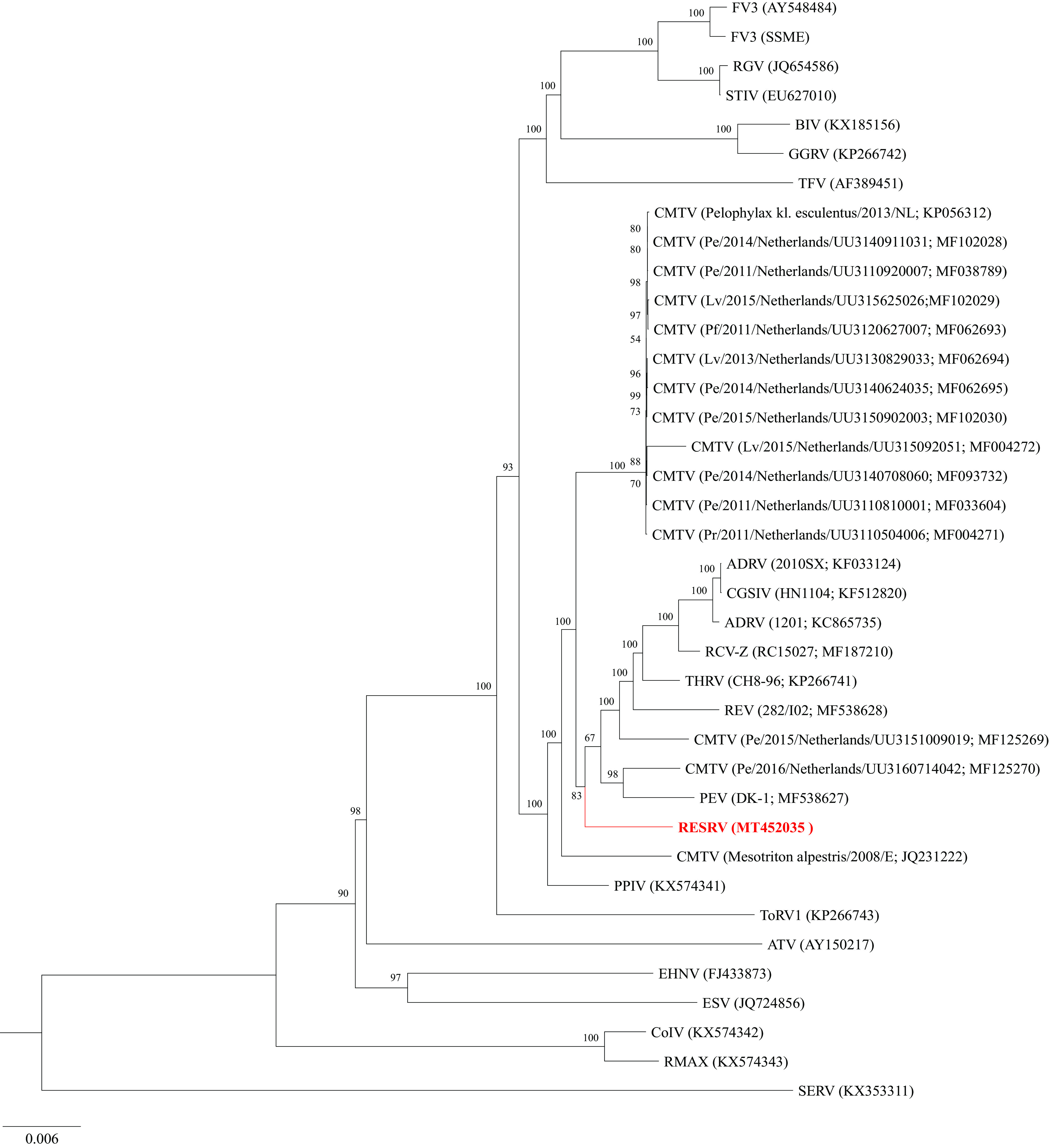
Maximum-likelihood phylogram depicting the relationship of RESRV (in red) to 37 ranaviruses based on the concatenated genome-wide locally colinear block alignments. Isolate/strain identification (where available) and GenBank accession numbers are listed in parentheses. The bootstrap values are provided at each node.

The genome of the red-eared slider ranavirus (RESRV) was annotated using the Genome Annotation Transfer Utility with default parameters, and the CMTV strain PEV was used as the reference genome. Additional putative open reading frames (ORFs) were identified using GeneMarkS, and gene functions were predicted based on BLASTP searches against the NCBI GenBank nonredundant protein sequence database. A total of 101 putative ORFs were predicted in RESRV, compared to strains of CMTV predicted to encode between 98 and 112 ORFs (5–12). An analysis of locally colinear blocks (LCB) in Mauve, with default parameters, revealed that the RESRV displays the same genome arrangement as other strains of CMTV (data not shown). The genome-wide LCB alignments were concatenated in Geneious v10.2.6 (13) and used in a maximum-likelihood (ML) analysis in IQ-Tree (http://iqtree.cibiv.univie.ac.at) with default parameters and 1,000 bootstrap replicates. The resulting ML tree supported the Polish RESRV as a strain of CMTV (Fig. 1).

Members of the genus *Ranavirus* (family *Iridoviridae*) are globally emerging viruses that have been reported in wild and captive populations of ectothermic vertebrates (14). Although previous experimental challenge studies demonstrated that red-eared sliders are susceptible to frog virus 3 (15, 16), our study confirms the susceptibility of a free-ranging RES to CMTV. Thus, this invasive chelonian represents a new host for CMTV and may potentially spread ranaviruses into native populations of fish, amphibians, and reptiles. Ethical approval for turtle trapping and euthanasia was not required according to the local bioethical committee at the University of Life Sciences in Lublin, Poland (statement of 14 October 2014).

### Data availability.

The genome sequence and raw sequence data for RESRV have been deposited in the NCBI GenBank and Sequence Read Archive (SRA) databases under accession no. MT452035 and SRX8622342, respectively.

## References

[1] European Environmental Agency. 2014 Regulation no. 1143/2014 of the European Parliament and of the council of 22 October 2014 on the prevention and management of the introduction and spread of invasive alien species. https://www.eea.europa.eu/policy-documents/ec-2014-regulation-eu-no.

[2] Johnson AJ, Pessier AP, Wellehan JFX, Childress A, Norton TM, Stedman NL, Bloom DC, Belzer W, Titus VR, Wagner R, Brooks JW, Spratt J, Jacobson ER 2008 Ranavirus infection of free-ranging and captive box turtles and tortoises in the United States. J Wildl Dis 44:851–863. doi:10.7589/0090-3558-44.4.851. 18957641

[3] Stilwell NK, Whittington RJ, Hick PM, Becker JA, Ariel E, van Beurden S, Vendramin N, Olesen NJ, Waltzek TB 2018 Partial validation of a TaqMan real-time quantitative PCR for the detection of ranaviruses. Dis Aquat Org 128:105–116. doi:10.3354/dao03214. 29733025

[4] Bankevich A, Nurk S, Antipov D, Gurevich AA, Dvorkin M, Kulikov AS, Lesin VM, Nikolenko SI, Pham S, Prjibelski AD, Pyshkin AV, Sirotkin AV, Vyahhi N, Tesler G, Alekseyev MA, Pevzner PA 2012 SPAdes: a new genome assembly algorithm and its applications to single-cell sequencing. J Comput Biol 19:455–477. doi:10.1089/cmb.2012.0021. 22506599 PMC3342519

[5] Ariel E, Subramaniam K, Imnoi K, Sriwanayos P, Ahasan MS, Olesen NJ, Amedeo M, Toffan A, Waltzek TB 2017 Genomic sequencing of ranaviruses isolated from edible frogs (*Pelophylax esculentus*). Genome Announc 5:e01015-17. doi:10.1128/genomeA.01015-17. 28935748 PMC5609427

[6] Chen Z, Gui J, Gao X, Pei C, Hong Y, Zhang Q 2013 Genome architecture changes and major gene variations of *Andrias davidianus* ranavirus (ADRV). Vet Res 44:101. doi:10.1186/1297-9716-44-101. 24143877 PMC4015033

[7] Claytor SC, Subramaniam K, Landrau-Giovannetti N, Chinchar VG, Gray MJ, Miller DL, Mavian C, Salemi M, Wisely S, Waltzek TB 2017 Ranavirus phylogenomics: signatures of recombination and inversions among bullfrog ranaculture isolates. Virology 511:330–343. doi:10.1016/j.virol.2017.07.028. 28803676

[8] Holopainen R, Subramaniam K, Steckler NK, Claytor SC, Ariel E, Waltzek TB 2016 Genome sequence of a ranavirus isolated from pike-perch *Sander lucioperca*. Genome Announc 4:e01295-16. doi:10.1128/genomeA.01295-16. 27856591 PMC5114383

[9] Saucedo B, Hughes J, Spitzen-van der Sluijs A, Kruithof N, Schills M, Rijks JM, Jacinto-Maldonado M, Suarez N, Haenen OLM, Voorbergen-Laarman M, van den Broek J, Gilbert M, Gröne A, van Beurden SJ, Verheije MH 2018 Ranavirus genotypes in the Netherlands and their potential association with virulence in water frogs (*Pelophylax* spp.). Emerg Microbes Infect 7:56. doi:10.1038/s41426-018-0058-5. 29615625 PMC5882854

[10] Stöhr AC, López-Bueno A, Blahak S, Caeiro MF, Rosa GM, Alves de Matos AP, Martel A, Alejo A, Marschang RE 2015 Phylogeny and differentiation of reptilian and amphibian ranaviruses detected in Europe. PLoS One 10:e0118633. doi:10.1371/journal.pone.0118633. 25706285 PMC4338083

[11] Mavian C, López-Bueno A, Balseiro A, Casais R, Alcamí A, Alejo A 2012 The genome sequence of the emerging common midwife toad virus identifies an evolutionary intermediate within ranaviruses. J Virol 86:3617–3625. doi:10.1128/JVI.07108-11. 22301140 PMC3302492

[12] van Beurden SJ, Hughes J, Saucedo B, Rijks J, Kik M, Haenen OLM, Engelsma MY, Gröne A, Verheije MH, Wilkie G 2014 Complete genome sequence of a common midwife toad virus-like ranavirus associated with mass mortalities in wild amphibians in the Netherlands. Genome Announc 2:e01293-14. doi:10.1128/genomeA.01293-14. 25540340 PMC4276818

[13] Duffus ALJ, Waltzek TB, Stöhr AC, Allender MC, Gotesman M, Whittington RJ, Hick P, Hines MK, Marschang RE 2015 Distribution and host range of ranaviruses, p 9–57. *In* Gray MJ, Chinchar VG (ed), Ranaviruses: lethal pathogens of ectothermic vertebrates. Springer, Cham, Switzerland.

[14] Kearse M, Moir R, Wilson A, Stones-Havas S, Cheung M, Sturrock S, Buxton S, Cooper A, Markowitz S, Duran C, Thierer T, Ashton B, Meintjes P, Drummond A 2012 Geneious Basic: an integrated and extendable desktop software platform for the organization and analysis of sequence data. Bioinformatics 28:1647–1649. doi:10.1093/bioinformatics/bts199. 22543367 PMC3371832

[15] Johnson AJ, Pessier AP, Jacobson ER 2007 Experimental transmission and induction of ranaviral disease in Western Ornate box turtles (*Terrapene ornata ornata*) and red-eared sliders (*Trachemys scripta elegans*). Vet Pathol 44:285–297. doi:10.1354/vp.44-3-285. 17491069

[16] Allender MC, Mitchell MA, Torres T, Sekowska J, Driskell EA 2013 Pathogenicity of frog virus 3-like virus in red-eared slider turtles (*Trachemys scripta elegans*) at two environmental temperatures. J Comp Pathol 149:356–367. doi:10.1016/j.jcpa.2013.01.007. 23582975

